# Biological Bases of Immune-Related Adverse Events and Potential Crosslinks With Immunogenic Effects of Radiation

**DOI:** 10.3389/fphar.2021.746853

**Published:** 2021-11-01

**Authors:** Lilia Bardoscia, Nadia Pasinetti, Luca Triggiani, Salvatore Cozzi, Angela Sardaro

**Affiliations:** ^1^ Radiation Oncology Unit, S. Luca Hospital, Healthcare Company Tuscany Nord Ovest, Lucca, Italy; ^2^ Radiation Oncology Department, ASST Valcamonica Esine and University of Brescia, Brescia, Italy; ^3^ Department of Radiation Oncology, University and Spedali Civili Hospital, Brescia, Italy; ^4^ Radiotherapy Unit, Clinical Cancer Centre, AUSL-IRCCS, Reggio Emilia, Italy; ^5^ Interdisciplinary Department of Medicine, Section of Radiology and Radiation Oncology, University of Bari “Aldo Moro”, Bari, Italy

**Keywords:** immune checkpoint inhibition, immune-related adverse events, self-tolerance, anticancer immune response, cytokines, commensal microbiome, radiotherapy, stereotactic body radiation therapy

## Abstract

Immune checkpoint inhibitors have gained an established role in the treatment of different tumors. Indeed, their use has dramatically changed the landscape of cancer care, especially for tumor types traditionally known to have poor outcomes. However, stimulating anticancer immune responses may also elicit an unusual pattern of immune-related adverse events (irAEs), different from those of conventional chemotherapy, likely due to a self-tolerance impairment featuring the production of autoreactive lymphocytes and autoantibodies, or a non-specific autoinflammatory reaction. Ionizing radiation has proven to promote both positive pro-inflammatory and immunostimolatory activities, and negative anti-inflammatory and immunosuppressive mechanisms, as a result of cross-linked interactions among radiation dose, the tumor microenvironment and the host genetic predisposition. Several publications argue in favor of combining immunotherapy and a broad range of radiation schedules, based on the recent evidence of superior treatment responses and patient survival. The synergistic modulation of the immune response by radiation therapy and immunotherapeutics, particularly those manipulating T-cell activation, may also affect the type and severity of irAEs, suggesting a relationship between the positive antitumor and adverse autoimmune effects of these agents. As yet, information on factors that may help to predict immune toxicity is still lacking. The aim of our work is to provide an overview of the biological mechanisms underlying irAEs and possible crosslinks with radiation-induced anticancer immune responses. We believe such an overview may support the optimization of immunotherapy and radiotherapy as essential components of multimodal anticancer therapeutic approaches. Challenges in translating these to clinical practice are discussed.

## Introduction

The advent of immune checkpoint inhibitors (ICIs) has dramatically changed the landscape of cancer care, since immunotherapeutic strategies are emerging as potentially curative systemic therapy for several tumors, especially for tumor types traditionally known to have poor outcomes. Treatment with a fully human anti- Cytotoxic T-lymphocyte–associated protein 4 (CTLA-4) antibody has been associated with long-lasting responses in several hematologic malignancies (Alatrash et al., 2016), and a high proportion of durable, complete responses in patients with advanced metastatic melanoma (Ascierto et al., 2015). Anti- Programmed cell death 1 (PD-1) or programmed death-ligand 1 (PD-L1) blocking antibodies have shown objective responses in a variety of solid tumors including melanoma, lung cancer, prostate cancer, breast cancer, ovarian cancer, head and neck cancer, and a subset of colorectal cancers (De Felice et al., 2015; El-Osta et al., 2016; Gong et al., 2016; Lynch and Murphy, 2016; Moskovitz et al., 2018; Calles et al., 2019). Immune checkpoint blockers have been demonstrated to enhance durable disease responses in both early and advanced tumor settings, alone or in combined strategies, as well as improving or retaining the patient’s quality of life (Porcu et al., 2019). However, although ICIs are globally less toxic than conventional chemotherapy agents, stimulating anticancer immune responses may also elicit an unusual pattern of immune-related adverse events (irAEs), unlike those of conventional chemotherapy, likely due to a self-tolerance impairment featuring the production of autoreactive lymphocytes and autoantibodies, infiltration of activated T-cells into normal tissues, or a non-specific autoinflammatory reaction (Hofman, 2019). Combination strategies (PD-1 or PD-L1 agents with other immunotherapy agents such as anti-CTLA-4 antibodies, molecular targeted therapy, vaccines, chemotherapies, radiotherapy, or chemoradiotherapies) may improve outcomes, as supported by recent evidence of superior treatment responses and patient survival. However, although multimodal therapies have the potential to overcome primary or acquired therapy resistance, they are also more likely to increase the rate of more severe and/or new adverse events (Barber, 2019). Immune-related side effects most commonly affect the skin, gastrointestinal tract, lungs, thyroid, pituitary, adrenal glands, and musculoskeletal system, while the nervous, renal, hematologic, ocular, and cardiovascular systems are less frequently involved (Barber, 2019). In particular, gastrointestinal and brain toxicity is more common with anti-CTLA-4 drugs, while hypothyroidism, hepatotoxicity and pneumonitis are more frequent in cases of anti-PD-1-targeted therapies (Waldman et al., 2020). A recent meta-analysis of trial data sets estimated a very wide range of incidence of irAEs, occurring in 15–90% of patients. Serious irAEs were reported in 30% of patients treated with anti-CTLA-4 and 15% with anti-PD-1 inhibitors (Hodi et al., 2010; Michot et al., 2016).

It is of critical importance to gain a good understanding of the different manifestations of irAEs in order to ensure early detection and proper management of patients treated with immunotherapy (Porcu et al., 2019). At present, irAEs are challenging both to diagnose and to treat. Several factors may influence the development of irAEs during treatment with ICIs, including the cancer type, molecular target (CTLA-4 or PD-1/PD-L1), single agent administration or combined checkpoint blockade and/or sequential use of different types of ICIs. Treatment duration and prior chemotherapy also likely influence immunotherapy tolerance, together with underlying host factors such as the patient’s age, genetic predisposition to autoimmunity and/or pre-existing autoimmune disorders, and the host microbiome (Weinmann and Pisetsky, 2019). However, basic and translational research on irAEs is still limited. Information on factors that may help to predict immune toxicity is still lacking, nor are the pathogenic mechanisms of irAEs yet fully understood. Since not all patients with pre-existing autoimmune diseases suffer an exacerbation of their underlying condition upon ICI prescription, it is conceivable that the pathogenesis of irAEs is a far more complex matter than simple breaches in the immune tolerance mechanisms alone (Esfahani et al., 2020a).

The aim of our work was to provide an overview of the current evidence on the biological bases underlying irAEs, and to speculate about possible crosslinks with radiation-induced anticancer immune responses.

## The Balance Between Self Tolerance and Anticancer Immune Response

Immune homeostasis acts to prevent autoimmune disease, and the CTLA-4 and PD-1 pathways are vital for this balance, but also involved in anticancer immune response exhaustion and suppression. In fact, the lack of CTLA-4 produces extensive infiltration of activated lymphocytes into lymph nodes, the spleen and thymus in murine models. Lymphocytes infiltration has also been observed in the heart, lung, liver and pancreas, but not in the kidney, together with high antibody levels (Weinmann and Pisetsky, 2019).

Recent studies indicate that irAEs may be the result of potential cross-reactivity between tumor and self, underlying collateral damage due to cytokine-induced inflammation, antigen-specific T-cell responses, autoantibodies, B cells (Young et al., 2018; Khan and Gerber, 2020).

A complex network of cell surface receptors and signaling pathways strictly regulates the balance between self tolerance and anticancer immune responses. ICIs improve the intensity and duration of the host immune response against tumor cells by blocking inhibitory signals. Such effects can shift immune responses away from tolerance in favor of an activated state in which the immune system reacts against both tumor antigens and antigens on healthy tissues, thus triggering irAEs (Esfahani et al., 2019).

## Proposed Immunopathogenic Mechanisms for irAEs

**FIGURE 1:**
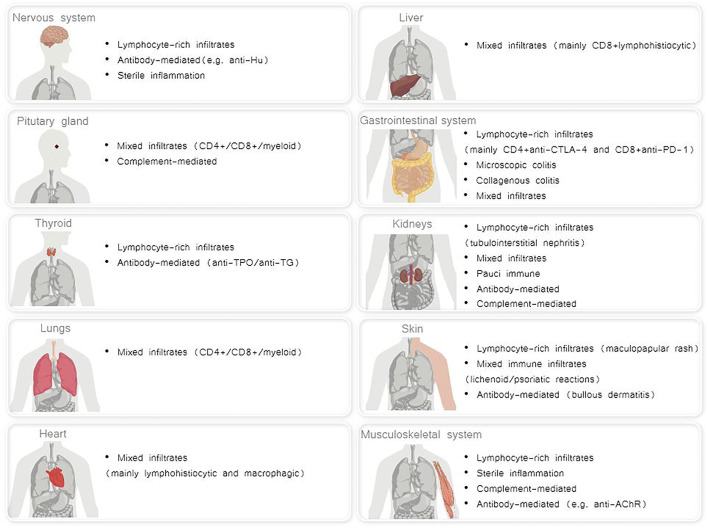
Distinct histopathological phenotypes of irAEs per each affected organ (Albarel et al., 2019; Esfahani et al., 2020a; El Sabbagh et al., 2020; Khan and Gerber, 2020).

A broad range of mechanisms underlying irAES has been described, that differ for each affected organ. It is conceivable that distinct immunopathogenic mechanisms result in irAEs, which, in turn, may lead to a variety of distinct histopathological phenotypes in each affected organ (Esfahani et al., 2020a) (Figure 1).

### Autoreactive T-Cells and B-Cells

Currently, irAEs are believed to reflect an exaggerated host immune reaction (Naqash et al., 2019). The role of both auto-reactive T- and B-cells, together with antibody-mediated processes, and a decreased T-regulatory phenotype, has been investigated (Petrelli et al., 2018; Esfahani et al., 2020a). The diversification and sub-compartmental expansion of lymphocytes induced by ICIs in secondary lymphoid organs, peripheral tissues and blood has been hypothesized to promote the development of autoreactive T cells and B cells (Esfahani et al., 2020a). Emerging data on patients developing irAEs support a cross-reactivity of T-cell clones with antigens and/or epitopes shared between tumor cells and healthy tissue (Berner et al., 2019; Rapisuwon et al., 2019). Another main feature may be the loss of naive T cells and the accumulation of overactive memory T-cells invading peripheral organs, triggering inflammatory damage (Waldman et al., 2020). Modulation of the regulatory T-cells (Treg) stability may also contribute to the balance between antitumor immunity and irAEs, since this subset of T-cells is involved in the exhaustion of the immune response. Targeting Treg-derived EZH2 or NRP1 genes, both of which are highly expressed in tumor-infiltrating Tregs, has been demonstrated to bolster the production of proinflammatory cytokines, hence producing antitumor immunity without autoimmune consequences (Overacre-Delgoffe et al., 2017; Wang et al., 2018a). Depletion of FoxP3+ Treg also seemed to worsen liver toxicity in murine models (Bartkowiak et al., 2018). Of note, ongoing disease responses despite ICI discontinuation due to irAEs have been described. The potential cross-reactivity of ICI-induced persistent CD8^+^ T effector memory subsets against tumor cells and normal tissues may be a plausible explanation for the durable benefit gained with ICIs. Moreover, anti-PD-1 antibodies are known to have a T-cells receptor occupancy of >2 months, and a half-life that spans 3–4 weeks, a steady state concentration being achieved within 19 weeks (Naqash et al., 2019). However, the varying populations of initial priming T-cells targeted by ICIs may influence the severity and time to onset of irAEs (Milling et al., 2017). A trend to higher frequencies of CD8^+^ T cells in both newly detected clones and increased preexisting clonotypes, as compared to CD4^+^ T cells, has also been reported in patients developing irAEs (Oh et al., 2017). The depletion of Treg at tumor sites and increase of peripheral CD8^+^ T effector cells have been hypothesized to drive irAEs, induced by CTLA-4 blocking antibodies (Milling et al., 2017). In particular, anti-CTLA-4 antibodies of the IgG2a isotype seemed to be capable of engaging Fcγ receptors expressed by tumor-associated macrophages within the tumor microenvironment in mouse models, hence to mediate an antitumor activity via selective elimination of intratumoral T cell populations, particularly Tregs (Bulliard et al., 2013; Simpson et al., 2013). Instead, other isotypes expanded both Treg and effector T-cells at the periphery (Selby et al., 2013). Overall, Treg depletion may lead to increased lymphocytes and leukocytes infiltration into the mucosal linings of organs including the lungs and gastrointestinal tract (Milling et al., 2017).

The role of B cells in the pathogenesis of irAEs is less well characterized than that of T-cells. Firstly, humoral immunity may play a role in some forms of irAEs (Young et al., 2018). Pathogenic autoantibodies, in turn, may be generated as a result of nascent autoreactive T cells and B cells that are able to escape central tolerance (Esfahani et al., 2020a). Supporting this concept, autoantibodies have been reported in some cases of ICI-induced type 1 diabetes, thyroiditis and hypothyroidism, and rheumatic disorders, but whether these antibodies represent a pre-existing immune substrate or are the consequence of ICI-induced self-tolerance alterations remains unknown (Esfahani et al., 2019). Antibodies to islet cell antigens and glutamic acid decardoxylase-65 were also reported to be associated with ICI-induced diabetes (Weinmann and Pisetsky, 2019). In PD-1 knockout mice, there is early evidence of the development of a lupus-like disease, with glomerulonephritis and renal deposition of IgG3 and C3, inflammatory arthritis in the majority of cases, and autoimmune dilated cardiomyopathy with severe heart failure (Nishimura et al., 1999; Nishimura et al., 2001). Conversely, autoantibody positivity for islet-associated antigens has been found in less than 50% of cases at diagnosis, versus 90% in conventional type 1 diabetes. Similarly, rheumatological irAEs have been associated with negative rheumatoid factor and cyclic citrullinated protein antibodies, and frequently low antinuclear antibodies at presentation. On the contrary, thyroid antibodies have been observed in cases of anti-PD-1-induced thyroid irAEs in non–small cell lung cancer (Stamatouli et al., 2018; Kurimoto et al., 2020). Nevertheless, the development of antinuclear antibodies or a lupus-like disease is not reported as a prominent feature of irAEs in the available literature. It is possible that a distinct pattern of B cell expression occurs after CTLA-4 inhibition (Weinmann and Pisetsky, 2019). In a series of 127 patients treated with ICIs by de Moel *et al.*,19.2% of them, who were antibody negative before treatment, developed new autoantibodies, more frequently anti-TPO (thyroperoxidase) and anti-TG (thyroglobulin), but the relationship between the emergence of autoantibodies and irAEs was not statistically significant. irAEs were reported in 78.9% of autoantibody-positive patients, compared to 57.5% of patients who were autoantibody negative before starting ICI therapy; thyroid dysfunction was observed in 44.4% of patients who exhibited anti-thyroid antibodies during anti-CTLA-4 therapy and then received anti-PD-1 ICI (de Moel et al., 2019).

Secondly, aberrations in various B-cell subsets have been observed (Dubrot et al., 2010). Heterozygous germline mutations in the gene encoding CTLA-4 have been associated with a decreased absolute B cell count, switched memory B cell counts, and the expansion of the subset CD21_lo_ memory B cells, where low levels of CD21 are usually responsible for B cell exhaustion and chronic antigen exposure in humans, who are thus more likely to develop irAEs (Rakhmanov et al., 2009; Schwab et al., 2018; Esfahani et al., 2020a). Such findings, together with a restricted expression of PD-1 and increased IFNɣ signaling in CD21_lo_ B cells themselves, were also observed after combined immune checkpoint blockade (Kuehn et al., 2014). Combination ICI-therapy has also been shown to be associated with a significant decrease in the number of circulating B cells after treatment, but increased expression of immunoglobulin genes and number of plasmablasts, together with plasma levels of the chemokine CXCL13, a marker of germinal centre activation in humans. Such early changes in B cells seemed to be correlated with higher rates of grade 3 or higher irAEs at 6 months after immune checkpoint inhibition (Weinmann and Pisetsky, 2019), as well as an earlier time to onset and severity of immune toxicity, irrespective of the site. However, it is not known whether such reduced proportions of B cells may reflect an effect on antibody production (Das et al., 2018; Young et al., 2018; Weinmann and Pisetsky, 2019).

### Diversification of the T-Cell Repertoire

There is a growing interest in T-cell receptor (TCR) repertoire profiling of T lymphocytes in tissues and/or peripheral blood, as a possible means of predicting the onset and severity of irAEs, and/or the therapeutic response to ICIs (Hofman, 2019). For instance, patients treated with anti-CTLA-4 antibody for metastatic castration-resistant prostate cancer who developed irAEs showed a significant early diversification of the CD4^+^ and CD8^+^ T-cell repertoires, and the generation of self-reactive clonotypes within 2 weeks of treatment, whereas non-irAE patients did not (Esfahani et al., 2020a). The development of irAEs was associated with an increased number of new TCR clones and preexisting clonotypes following ipilimumab plus Granulocyte-macrophage colony-stimulating factor (GM-CSF) treatment, as compared with non-irAE patients (Oh et al., 2017). Pre-clinical evidence suggested that polyclonal T-cell expansion may cause an increased accumulation of mononuclear, cytotoxic cells in the portal areas of the liver, thus contributing to hepatocytes apoptosis and subsequent hepatitis (Niu et al., 2007; Dubrot et al., 2010). A recent study showed that immune toxicities elicited by CTLA-4 blockade were associated with early diversification of the T cell repertoire (Oh et al., 2017). In addition, alterations to the T-cell repertoire in response to ICIs have been reported to be correlated with both the therapeutic response and the severity of irAEs (Young et al., 2018). A different total number of productive T cell repertoire V b sequences in the complementarity determining region (CDR3) in patients with and without irAEs has also been reported, but a concomitant expansion of particular clones in the peripheral blood was not observed (Weinmann and Pisetsky, 2019).

Such findings suggest that irAEs may derive from a mobilization of large amounts of T cells, some of which are autoreactive to multiple antigens and gradually emerge after checkpoint blockade (Weinmann and Pisetsky, 2019). However, despite the detected early increase in TCR diversity in irAE patients, the actual toxicities usually manifested with variable kinetics: the skin was typically involved earliest, followed by the onset of colitis (after 1–3 administrations), and lastly of hepatitis and endocrinopathies (Oh et al., 2017; Hofman, 2019). This means that TCR repertoire diversification may represent only a part of the pathogenic mechanisms underlying toxicity, including possible additional repertoire changes that occur nearer in time to the actual onset of irAEs.

### Neutrophil, Eosinophil, Monocyte-derived Macrophages Activation

There is evidence of gastrointestinal side effects in cancer patients treated with anti-CTLA-4 antibody, associated with an increased expression of the biliary glycoprotein CECAM-1 and the cell surface glycoprotein CD177, which is directly correlated with neutrophil activation (Friedlander et al., 2018). Abnormal CTLA-4 recycling and subsequent lysosomal degradation have also been shown to contribute to toxicities, and possibly to a reduced drug effectiveness (Waldman et al., 2020).

Eosinophils have been reported to be related to the occurrence of irAEs, but seemed to have no impact on survival during treatment. Functional links between Interleukin (IL)-17 and eosinophils have been hypothesized, since eosinophils do not only act as effector cells but also drive the IL-23/IL-17 axis and contribute to the recruitment of inflammatory cells (Schindler et al., 2014). Indeed, increases in eosinophil counts and release of the pro-inflammatory cytokine IL-17 have been identified as predictive biomarkers of toxicity of ICIs, regardless of the affected organ (Waldman et al., 2020).

There is also evidence of monocyte-derived macrophages having a critical role in the PD-1/PD-L1 inhibitor-induced autoimmune diabetes. Hu and colleagues demonstrated that the high levels of IFN-γ produced by exhausted CD8^+^ lymphocytes promote the recruitment of monocyte-derived macrophages in mice. Such highly activated monocyte-derived macrophages acquire cytocidal activity against pancreatic islet β-cells, hence driving diabetogenesis, while their temporal depletion dramatically protected mice from β-cell damage. (Hu et al., 2020). Autopsy-examined patients treated with anti-CTLA-4 antibodies alone or in combination with PD-1/PD-L1 inhibitors confirmed predominant T-cell infiltrates in irAEs, but also a consistent inflammatory infiltration by type 2 macrophages (M2) and Treg depletion. Such findings further suggested that the progressive organ damage mediated by M2 and depletion of T-regs play an important role in the pathogenesis of irAEs (Mihic-Probst et al., 2020). The activation of peripheral monocytes has also proven to be involved in the pathogenesis of ICI-induced hepatitis. A recent analysis of phenotypic and transcriptional profiling of peripheral immune subsets of patients treated with ICIs and developing hepatitis showed the presence of activated monocytes and enhanced effector CD8^+^ T cells, compared to those without hepatitis and healthy controls. To support this, CD163^+^/CCR2^+^ macrophages and CD8^+^ T cells were found in liver inflammation infiltrate (Gudd et al., 2021).

Moreover, off-target effects of ICIs on non-haematopoietic cell lines bearing the target immune checkpoint ligand have been described in a preclinical model. In this regard, the CTLA-4 receptor is also expressed on hypothalamic and pituitary tissues, so that hypophysitis is particularly common with CTLA-4 antibodies and combination therapy, but such immune-related endocrine side effect has also been observed during anti-PD-1 use, although less frequently (Albarel et al., 2019; Esfahani et al., 2020a).

### Adaptive and Innate Immunity

The collateral damage to non-transformed tissues distributed in and around the tumor microenvironment induced by adaptive and innate anti-tumor immune responses is presumed to facilitate the host autoimmunity. An interesting model speculated on molecular mimicry leading to antigenic cross-presentation, or the release of self-antigens, which favor the activation of autoreactive T or B cells, although no evidence of specific, self-antigen- directed adaptive immune responses is available in literature (Rojas et al., 2018). Cells of both the adaptive and the innate immune systems produce IL-17, which represents a cross-link between immune and non-immune cellular activities. This pathway inhibits the production of Treg, thus reversing anti-cancer immune-response exhaustion (Esfahani et al., 2020a). The balance between IL-12 and IL-23 has been identified as an important regulator of carcinogenesis and autoimmunity: IL-12 promotes T helper (Th) 1 cells-mediated innate and adaptive immune responses, while IL-23 has a key role in Th17-mediated adaptive immunity (via IL-17 production) (Ngiow et al., 2013).

Interferons (type I IFNα, IFNβ, IFNε, and IFNω) and type II interferon (IFNɣ) also drive innate and adaptive immune responses. Interferons mostly act through the JAK–STAT signal transduction pathway. In the context of anticancer immune responses, an intact IFNɣ axis is essential for the expression of PD-1 and PD- L1, hence in regulating responses to ICIs (Esfahani et al., 2020a). Likewise, the mTOR pathway modulates both innate and adaptive immune responses (Powell et al., 2012). Combining mTOR inhibition and anti-PD-1 therapy in cancer patients requiring ICIs, with a history of solid organ transplantation, ensured allograft tolerance by abating the threshold for innate and adaptive immune response activation, while preserving the IFNγ signaling required for the efficacy of anti- PD-1 therapy (Esfahani et al., 2020a).

### Autoinflammatory Mechanisms

Integrin-mediated T cells extravasation into peripheral tissues is known to be responsible for tissue inflammation. In the cancer immunotherapy setting, the release of inflammatory cytokines from immune cells may potentially play an important role in mediating irAEs (Esfahani et al., 2020a).

IL-1 has a crucial role in autoinflammatory diseases (Sutton et al., 2019). This cytokine is predominantly involved in the innate immune response, secreted by macrophages and monocytes. Higher levels of IL-1β and lower levels of IL-1 receptor antagonist (IL-1RA) were found in myeloid immune cells of patients with solid tumors treated with ICIs who developed immune-related pneumonitis. Elevated blood levels of IL-1 have also been detected in patients with melanoma who suffered a wide spectrum of irAEs (Lim et al., 2019; Suresh et al., 2019).

Cytokines like tumor necrosis factor (TNF) α and IL-17 are known to enable tumor surveillance, but they can also mediate autoimmune reactions. In fact, the use of IL-17 monoclonal antibody for the treatment of immune-related colitis and psoriasis has been reported to reduce immune toxicities, but also induce tumor escape. Conversely, TNFα blockade has been demonstrated to uncouple the efficacy and toxicity of ICIs targeting PD-1 in the treatment of ICI-related colitis in animal models (Esfahani et al., 2020a). Elevated baseline circulating levels of TNFα have also been described as a biomarker for the subsequent development of irAEs (Head et al., 2019a). The CD8^+^ T-cell production induced by TNFα, in turn, has been demonstrated to be critical in the development of splenomegaly, lymphadenopathy, hepatomegaly, and hepatitis (Milling et al., 2017).

IFNɣ and type I interferons were reported to contribute to the expansion of blood cells and mislocalization of T-cells (Milling et al., 2017). Serum levels of IFNɣ inducible chemokines CXCL-9 and CXCL-10 have also been shown to be correlated with the development of irAEs (Khan and Gerber, 2020). Preliminary evidence suggests that IL-27 produced by anti-CD137 stimulated myeloid subsets to mediate the recruitment and activation of liver damaging T cells (Milling et al., 2017).

Increased circulating levels of IL-6 and C-reactive protein (CRP) (whose expression is directly dependent on IL-6 during ICI administration) have been recorded in patients with different tumor types developing a broad range of irAEs, and a synergistic antitumor activity has been demonstrated in mouse models combining ICIs with inhibition of the IL-6 axis (Esfahani et al., 2020a). Evidence of elevated levels of IL-6 and CRP in patients with irAEs, as compared to baseline pre-treatment levels, as well as elevations in IL-17 and IL-10 levels, indirectly supported the induction by the altered cytokine physiology of an immune dysregulation during irAEs. However, these results need to be interpreted with caution, given the lack of prospective validation to account for confounding factors (e.g., potential infection) which may contribute to cytokines dysregulation (Naqash et al., 2019).

### Genetic Susceptibilities

Current analyses of irAEs lack studies on epigenetics, so the impact of genetic susceptibilities on the development of irAEs remains poorly explored. Distinct human leukocyte antigen (HLA) haplotypes, and polymorphisms in immunoregulatory genes such as CTLA-4 and PD-1 have been associated with a variety of classical autoimmune diseases, and likely play an important role in the development of irAEs (e.g., HLA-DQ8 and HLA-DR53 for lymphocytic hypophysitis), and the predominance of HLA-DR4 among patients treated with PD-1- or PD-L1-directed ICIs developing autoimmune insulin-dependent diabetes (Khan et al., 2018)). Conversely, the effect of genetic variants of CTLA-4, PD-1, and PD-L1 on the risk or severity of irAEs is still little understood (Esfahani et al., 2019).

### The Role of the Microbiome

The human microbiome has been historically defined as the set of microbes, mainly bacteria, and their genes in the human body, involved in the regulation of key metabolic pathways and immunomodulation (Joseph et al., 2020).

Recently, the microbiome has been reported to modulate irAEs, besides priming the immune response to ICIs. Specific microbial metabolic pathways, such as polyamine transport and vitamin B synthesis, were found to be predictive for resistance to colitis induced by CTLA-4 blockade, as well as a Bacteroidetes-rich phylotype (Esfahani et al., 2020b). The bacterial composition of the microbiome may influence differentiation of the immune lineage towards pro-inflammatory or regulatory cell subtypes, exacerbating the pro-inflammatory effects during ICI administration. In this regard, commensal *Bacteroides fragilis* have been shown to facilitate the development of Treg cells, producing anti-inflammatory IL-10, while these segmented filamentous bacteria promote the differentiation and generation of Th17 cells and the related pro-inflammatory pathway in the small intestine (Al-Qadami et al., 2019).

Not surprisingly, the most common irAEs involve organs with a rich content of commensal organisms, such as the skin, colon and lungs (Esfahani et al., 2020a). Accordingly, exposure to antibiotics has been reported to worsen survival outcomes, and increase the incidence and severity of irAEs (Abu-Sbeih et al., 2019; Elkrief et al., 2019). Nevertheless, studies of the effects of the microbiome on ICI efficacy and toxicity cannot ignore concomitant factors which may affect the integrity of the commensal microbiome composition, such as exposure to antibiotics or other drugs (proton-pump inhibitors, antipsychotics, antimetabolites), radiation therapy (RT), and diet (Dubin et al., 2016).

## Radiotherapy and Immunotherapy

Radiotherapy and conventional cancer chemotherapy are the standard of care, and are being readily incorporated into combined approaches with ICIs, in view of the pre-clinical evidence of a synergistic immune stimulation against cancer cells and the promising results in clinical trials (Eckert et al., 2018).

Subsets of T cells are known to have a role in the normal tissue damage induced by ionizing radiation, acquiring a pro-inflammatory and pro-fibrotic phenotype during both the acute and chronic phases of the tissue response (Citrin and Mitchell, 2017). For this reason, patients with autoimmune diseases, such as interstitial lung disease, inflammatory bowel disease, and connective tissue diseases may supposedly have a predisposition to radiation toxicity (Hwang et al., 2018).

In recent years, the mechanistic rationale for combining RT and ICIs has been detailed in various reports (Patel and Minn, 2018; Jagodinsky et al., 2020). The immunogenic effects of such genotoxic cancer therapies have been hypothesized to be strongly influenced by the adaptive immunity, as mainly expressed by an enhanced pattern recognition receptors (PRR) signaling (Patel and Minn, 2018). In this regard, ionizing radiation acts *via* the cGAS-STING signaling pathway in dendritic cells, with subsequent type I IFN production that, in turn, amplifies immune recognition and ultimately, T-cell recruitment. Radiation-induced diversification of the intratumoral TCR repertoire, improved antigen processing and increased Major Histocompatibility Complex I (MHC-I) expression have also been observed in experimental models, as well as in cancer patients undergoing RT (Muraro et al., 2017; Zhang et al., 2017). Cancer irradiation is also well known to reactivate the expression of some tumor-associated antigens and contemporarily promote the accumulation of tumor neoantigens on the cell surface, thereby changing the tumor cell phenotype. On the other hand, high-dose RT can also drive immune-suppressive Treg expansion and CD8^+^ T-cells exhaustion (Gandhi et al., 2015). The addition of a dual checkpoint blockade (anti-CTLA-4 inhibiting Treg, and anti-PD-1/PD-L1 enabling prolonged cytotoxic immunity) may reverse such negative feedback pathways, and enhance RT-induced immune-killing (Jagodinsky et al., 2020). Moreover, combining radiation with anti-CTLA-4 blockade has been shown not only to produce regression of the irradiated tumor, but also a response in out-of-field (*abscopal* effect) tumor sites, in mice as well as in some case reports (Patel and Minn, 2018).

Several publications have reported hypofractionated RT to be safe and effective in combination with ICIs. Fractionation allows the delivery of therapeutic, high-dose radiation to the tumor mass while sparing the surrounding normal tissues (Martin et al., 2018). Symptomatic radiation necrosis has been reported in patients treated with stereotactic radiosurgery (SRS) plus ICI therapy, especially melanoma (Martin et al., 2018), while murine models first revealed that fractionated stereotactic body radiotherapy (SBRT) schedules enable a more sustained *abscopal* effect than single 20 Gy fraction RT in combination with anti-CTLA-4 antibody (Dewan et al., 2009). In this setting, the magnitude of the immune response elicited by single, high-dose RT was supposed to be insufficient at controlling the tumor burden outside the radiation field, while fractionated SBRT selectively induced several IFN-related genes, suggesting the ability of sublethal radiation doses to improve tumor cells adjuvanticity (Dewan et al., 2009). Selective upregulation of IFN-related genes by fractionated (2 Gy × 5 fractions) but not single dose radiation has been reported in human breast, prostate, and glioma tumor culture cells (Tsai et al., 2007a). An *in vitro* comparison of the gene response of the prostate carcinoma cells to single dose and fractionated RT in mice also supported the role of the tumor microenvironment and the radiation schedule in modulating the pro-immunogenic effects of radiation (Tsai et al., 2007b). Actually, genome-wide gene expression analyses have been investigating the differences between the cross-priming induced by SRS and SBRT (John-Aryankalayil et al., 2010; Demaria and Formenti, 2012; Eckert et al., 2017).

Alongside the improved synergistic efficacy, reduced antitumor effects have been described in cases of ICIs plus RT administration for the treatment of large tumors (Eckert et al., 2018), together with preclinical evidence of conventional normofractionation to possibly have anti-immunogenic properties through the induction of Tregs (Kachikwu et al., 2011). Nevertheless, dynamic changes in murine models cannot fully mirror what may happen in human patients. T cells are notoriously radiosensitive, tumor-infiltrating T cells are unavoidably irradiated, especially during prolonged courses of radiation, and such vulnerability of leucocytes to RT may also be responsible for blood leucopenia and/or lymphopenia, thus compromising patients immune tolerability and anticancer immune response (Wang et al., 2018b). However, retrospective data and prospective clinical trials suggested that combining RT and immunotherapeutics is safe, and seemed to confirm the strong preclinical rationale (Jagodinsky et al., 2020; Vanneste et al., 2020).

Unfortunately, information on how RT can affect the risk, if present, of adverse events in cancer patients treated with ICIs, is still lacking in literature. A recent pooled analysis from the U.S. Food and Drug Administration showed overall similar rates of irAEs between the RT and no-RT groups [9,087 (35%) and 16,749 (65%) of the population under study, respectively], with numerically higher hematologic toxicities and pneumonitis in the former, and patients receiving RT more likely to develop grade 3–5 hematologic toxicities than those who did not (Anscher et al., 2020).

Whether or not patients, tumor and treatment parameters (type of ICI, timing, dose, fractionation and site of radiotherapy) may influence efficacy and safety of adding RT to ICIs is still unknown (Bang and Schoenfeld, 2019).

In some cases, the onset of irAEs may have a favorable impact on patients undergoing radioimmunotherapy. Recently, a prospective evaluation by Schweize *et al.* reported metastatic melanoma patients developing irAEs to have a significantly prolonged overall survival than those who did not in the whole population under study and the subgroup treated with RT, with no differences in irAEs rate between the RT group and non-RT group (Schweizer et al., 2020). A retrospective study on concurrent, palliative RT and a PD-1 inhibitor (usually 30–3 Gy/fraction using 3D conformal RT techniques) showed only 36% of grade 3 toxicities occurring within the radiation field, while all grade 4 and 5 toxicities occurred outside the field (Parker et al., 2018). Bang and colleagues retrospectively revealed neither differences in the overall rates of all grade (35%) and grade ≥3 toxicities (8%) with the addition of palliative RT to ICIs for the treatment of solid tumors (including melanoma, NSCLC and renal cancer), when delivering a median equivalent dose in 2 Gy fractions (EQD2Gy) of 40 Gy, nor correlations between the site of the target volume and increasing rate of any associated adverse event. On the contrary, there was an increased incidence of irAEs of any grade for EQD2Gy above median (50% vs 42% in the non-RT group; *p =* 0.01), and a trend towards a higher rate of irAEs of any grade for radiation delivery within 14 days of ICI (39% versus 23%; *p =* 0.06). However, no association was found between biologically effective radiation dose (BED) and severe irAEs, and statistical significance was lost when directly comparing patients receiving RT plus anti-PD-1 antibodies and RT plus anti-CTLA-4 therapy (Bang et al., 2017). Such findings were in line with a prospective phase I trial by Luke *et al.*, including patients receiving extracranial 30–50 Gy in 3–5 fractions followed by the initiation of a PD-L1 inhibitor within a week, but with evidence of an association between the site of radiotherapy and toxicities, too (Luke et al., 2018). The prospective study design, the use of higher radiation doses, and the relatively short interval between the end of RT and initiation of ICI may be a possible explanation for the latter difference. Therefore, the primary role of systemic interactions rather than local ones depending on the irradiated site in the emergence of toxicities from combination treatments may even be reasonably hypothesized (Hwang et al., 2018). To support this, the fractionation-corrected mean radiation dose delivered to the lungs (MLD) has recently been shown to significantly predict ≥ grade 2 radiation pneumonitis in a cohort of patients undergoing lung SBRT or hypofractionated RT after recovery of prior irAEs. In particular, a 5 Gy MLD was associated with a 50% risk of ≥grade 2 pulmonary toxicity, while 21 out of 26 patients (81%) with a MLD of >5 Gy developed ≥ grade 2 radiation pneumonitis (Shaverdian et al., 2020).

## Potential Markers Predictive for irAEs

To date, the detection of factors which may predict patients developing an irAEs is still a challenge.

Preexisting abnormal antibodies, such as rheumatoid factor (RF) or antinuclear antibody, were shown to be independently correlated with irAEs, but no significant correlation was observed between antibody expression levels and the severity of irAEs (Toi et al., 2019).

### Nonspecific biomarkers

A retrospective study reported increased circulating levels of C-reactive protein (CRP) in patients with ICI-induced pituitary inflammation, hepatitis, thyroiditis and autoimmune colitis (Abolhassani et al., 2019). Since the CRP represents an inflammatory acute phase reactant, its level is susceptible to a variety of acute or chronic infections, anti-infective and anti-inflammatory drugs and autoimmune diseases, so that an increase of CRP at least twice during the 2-week interval of ICI administration, together with low levels of procalcitonin, and no evidence of infection (culture and serology) have been suggested as predictors of irAEs (Abolhassani et al., 2019).

Patients with low serum albumin showed a higher risk to develop anti-PD-1-related pneumonitis, instead (Fukihara et al., 2019). Overall, the incidence of grade 3–4 irAEs, especially pneumonitis, has been shown to be associated with increased white blood cell counts and decreased relative lymphocyte counts (Fujisawa et al., 2017). The rate of anti-PD-1 antibodies-induced toxicity was previously suggested to be related to a higher baseline and increase in absolute lymphocyte counts, and absolute eosinophil counts after ICI administration (Diehl et al., 2017)**.** A low baseline proportion of peripheral blood CD4^+^ Tregs has been reported to be associated with anti-CTLA-4-related colitis (Shahabi et al., 2013), while eosinophils had already been found in biopsy specimens from patients with anti-CTLA-4-induced hepatitis, rash and colitis (Berman et al., 2010; Lacouture et al., 2014; Johncilla et al., 2015). Of note, the patient’s inflammatory state and a variety of non-cancer related conditions may primarily affect the blood cell count, nevertheless the additional myelosuppression eventually induced by chemotherapy and/or RT may contribute to the blood cell count impairment. (Jia et al., 2020).

Cytokines and chemokines represent other potential biomarkers for predicting irAEs. Baseline elevation of TNFα and IFN-α2 levels in melanoma patients blood samples collected prior to the start of ICI therapy has revealed to be associated with the development of higher grades of irAEs (60 and 44% of the analyzed samples, respectively), although no associations between immune markers and the number and type of ICI-induced side effects in individual patients were observed (Head et al., 2019b). Both low baseline levels, and high levels of IL-6 after ICIs have also been described to predict the occurrence of irAEs, such as Crohn’s disease (Atreya et al., 2000), psoriasiform dermatitis (Okiyama and Tanaka, 2017), and colitis (Valpione et al., 2018). The upregulation of plasma levels of CXCL9, CXCL10, CXCL11 and CXCL13 was closely related to the occurrence of irAEs, as well as significantly higher levels of CXCL2, CCL20, CXCL8 and CCL23 were found in patients with irAEs than in those without immune-related toxicities (Khan et al., 2018), but yet, no association with organ-specific irAEs has been observed. Lim *et al.* also showed that increased expression of colony-stimulating factor-1 (CSF-1), fractalkine, and IL families is closely related to severe irAEs (Lim et al., 2019).

Alternatively, evaluation of muscle quality on CT scan has been proposed as a potential marker able to predict the development of severe irAEs. Sarcopenia and low muscle attenuation were described as independent factors significantly associated with high-grade toxicities from anti-CTLA antibody in metastatic melanoma (Daly et al., 2017).

In the near future, high tumor mutation burden might be a promising, nonspecific biomarker of irAEs in vulnerable cancer patients. In this regard, a significant positive correlation during anti-PD-1 treatment was shown in a variety of solid tumors (Bomze et al., 2019), but no link with organ-specific irAEs, or evidence about other ICIs like PD-L1 and CTLA-4 inhibitors have been found. Therefore, such findings need to be further explored in large prospective clinical studies. Importantly, higher baseline levels of soluble CTLA-4 (sCTLA-4) have been proposed as possibly predictive of immune toxicity in melanoma patients treated with anti-CTLA-4 antibody, especially gastrointestinal irAEs (Pistillo et al., 2019), although to some extent, high levels of sCTLA-4 may also reflect tumor immune escape and high tumor burden (Ward et al., 2013).

### Organ-specific biomarkers

Specific disorders of the gut microbiome, such as the absence of Bacteroidetes and high levels of Firmicutes in stool samples have been reported with higher risk of developing immune-related colitis (Dubin et al., 2016; Chaput et al., 2017).

There is also evidence of some humoral factors as possible predictive biomarkers of gastrointestinal irAEs. Higher blood levels of neutrophil activation markers, mainly CD177, and CEACAM1, prior to the absolute neutrophil count have also been described in this type of patients (Shahabi et al., 2013). A retrospective study showed a close relationship between peripheral blood mRNA expression of CCL3, CCR3, IL-5, IL-8 and PTGS2 and immune-related diarrhoea, especially grade 2–4 diarrhea (Friedlander et al., 2018), while upregulation of IL-17 levels at baseline and 6 weeks after treatment with anti-CTLA-4 antibody was reported to correlate with grade 3 diarrhea and colitis in melanoma patients (Tarhini et al., 2015).

Increased levels of anti-CD74 autoantibodies after ICI therapy have been demonstrated to be notably correlated with immune-related pneumonia (Tahir et al., 2019).

Thyroid dysfunction, even destructive thyroiditis were reported to be significantly associated with high baseline levels of anti-TG and anti-TPO antibodies (Osorio et al., 2017; Kobayashi et al., 2018), as well as early increase (before 4 weeks) in blood thyroglobulin levels (Kurimoto et al., 2020). Besides, the baseline thyroid uptake of fluorodeoxyglucose 18F-FDG has shown to increase the risk of anti-PD-1-related thyroid toxicity (Yamauchi et al., 2019). All this considered, pre-treatment evaluation of anti-thyroid antibodies might help identify patients with a high risk of thyroidal irAEs and improve the clinical benefit of ICIs, supported by the possible role of functional imaging in monitoring the dynamic changes of thyroid 18F-FDG uptake.

Despite its rarity, anti-PD-1/PD-L1-related type 1 diabetes has also been associated with islet autoantibody, insulin autoantibody and islet antigen 2 antibody positivity in some cases (Stamatouli et al., 2018).

Elevated levels of anti-GNAL and anti-ITM2B autoantibodies have been reported in cancer patients developing immune-related hypophysitis (Tahir et al., 2019). Since GNAL and ITM2B are target proteins with an established role as signal transducers in the normal secretion of various pituitary hormones, such as Thyrotropin-releasing hormone (TRH) and Adrenocorticotropic hormone (ACTH) (Kilger et al., 2011; Peverelli et al., 2014), disorders of such pituitary hormone levels as a sign of impaired pituitary function may become another interesting field of study.

Circulating anti-conductive tissue autoantibodies (ACTA) were suggested as a possible predictive biomarker for ICI-related cardiotoxicity, together with the rise of troponins and BNP in some cases of myocarditis after ICI treatment (Läubli et al., 2015; Johnson et al., 2016).

Finally, a common genetic variant of HLA haplotype, HLAdrb1*11:01, has been observed in a retrospective study as related to itching, and may be a possible future biomarker predictive for skin toxicity (Hasan Ali et al., 2019), as well as HLA genotypes dominated by DR4 were described in patients with anti-PD-1/PD-L1-induced type 1 diabetes (Stamatouli et al., 2018). Such findings increase the credibility of susceptible HLA genotypes as potential markers to predict patients predisposition to autoimmune diabetes before or during ICI therapy.

## Discussion

The future of cancer immunotherapy might rely on combination therapies, including not only checkpoint inhibitors, but also personalized cancer vaccines and novel targeted therapies, e.g., against the tumor microenvironment, tumor glycosylation, and/or the commensal microbiome, alone or in combination with chemotherapy, radiation and/or chemoradiation (Eckert et al., 2017). Radiotherapy has proven to elicit both positive pro-inflammatory and immunostimolatory activities, and negative anti-inflammatory and immunosuppressive mechanisms, as a result of cross-linked interactions among radiation dose, the tumor microenvironment and the host genetic predisposition (Demaria and Formenti, 2012; Wennerberg et al., 2017). Ionizing radiation, characterized by the ability to stimulate the innate immune system, and thus indirectly adaptive immune responses and antigen release, induces immunogenic tumor cell death. This suggests the possibility of turning tumor cells into an *in situ* vaccine, exerting local tumor control, and possibly triggering an additional response at distant tumor sites (the so-called *abscopal* effect) (Formenti and Demaria, 2009; Wennerberg et al., 2017; Joseph et al., 2020).

However, the right combination of immunological adjuvants to induce an optimal immune activation remains a challenge. A correlation between autoimmunity and irAEs has been emerging more and more clearly. However, this is mostly based on case reports, while longitudinal immune profiling assessing the mechanistic profile of irAEs is lacking (Khan and Gerber, 2020).

The clinical spectrum of irAEs is extremely various, and may involve a single organ or, less frequently, several districts. Unfortunately, toxicity data are not always correctly reported in clinical trials. In fact, information on the time of onset, reversibility and management are often lacking (Weber et al., 2013).

**FIGURE 2:**
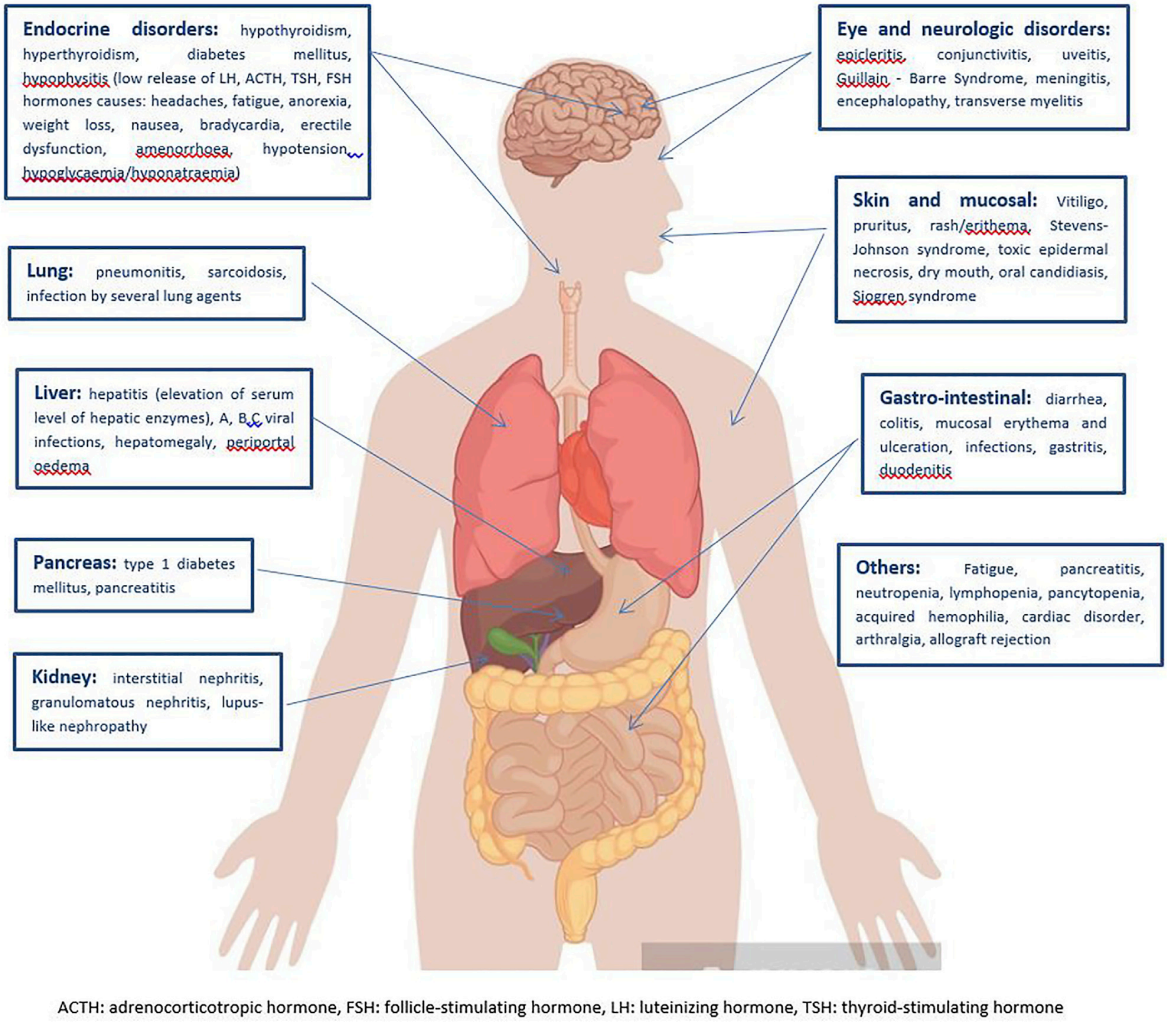
Clinical spectrum of irAEs per each affected organ (Michot et al., 2016; Martins et al., 2019).

Stratification of the main immune-related disorders organ-by-organ is reported in Figure 2.

Among the more common signs and symptoms of irAEs, fatigue is the typical main side effect of most medical treatments, including radiation, and ICIs make no difference. It is extremely difficult to understand the origin of such a non-specific symptom, which may be the result of very heterogeneous settings. To date, an effective coded treatment of fatigue does not exist, with often subsequent, unavoidable impairment of patients’ quality of life (Michot et al., 2016). The skin and mucous membranes are involved in 34–45% of immune-related toxicity, often with early onset in the first weeks of ICI administration. Vitiligo, pruritus, rash/erythema, dry mouth, oral candidiasis are the most frequent clinical presentations, while Stevens-Johnson Syndrome, toxic epidermal necrosis, Sjogren Syndrome are rarely reported. Topical emollients, oral antihistamine drugs and/or mild strength topical corticosteroids are usually sufficient to ensure a rapid symptom regression, but mild to high dose oral corticosteroids may be considered in case of severity (Haanen et al., 2018). Diarrhea, colitis, mucosal erythema and ulceration, infections, are well described for CTLA-4 inhibitors, less for anti-PD-1 antibodies. When basal antidiarrhoeal drugs, fluid and electrolyte supplementation do not lead to clinical remission, systemic corticosteroids or other additional immune suppressions with biological agents, such as infliximab or vedolizumab are required (Martins et al., 2019). Nearly 5–10% of patients receiving ICIs are also likely to develop endocrine irAEs. Among these, hypothyroidism is thought to occur more commonly than hyperthyroidism and hormone replacement therapy (i.e., Levothyroxine) should be prescribed. Hyperthyroidism spontaneously resolves in almost all cases (Haanen et al., 2018; Martins et al., 2019). Immune-related pneumonitis, including sarcoidosis and organizing inflammatory lung injury, commonly presents typical radiological features, that is ground-glass lesions and/or disseminated nodular infiltrates, which may be worsened by concurrent RT. High-dose oral steroids are the treatment of choice for such immune adverse events, but additional immune suppression with infliximab could be considered in case of refractory symptoms. Hepatitis has been described in 5–10% of cases, but it can arise up to 30% for combined treatments (Hwang et al., 2018; Shaverdian et al., 2020). Hepatitis is often asymptomatic, and just detected on routine blood monitoring of transaminases and bilirubin, whose persistent elevation longer than 1–2 weeks may need to be treated with mild to high dose oral corticosteroids, or, for refractory symptoms, mycophenolate mofetil for further immune suppression (even a liver biopsy may be considered) (Martins et al., 2019). Despite its rarity, renal toxicity may occur, especially for combined CTLA-4 and PD-1/PD-L1 inhibitors. Interstitial nephritis, granulomatous nephritis, lupus-like nephropathy are well treated with oral corticosteroids (Michot et al., 2016). Finally, a wide range of neurological events have been reported during ICI therapy, such as polyneuropathy, facial nerve palsy, demyelination, myasthenia gravis, Guillain-Barre’ syndrome, posterior reversible leukoencephalopathy, transverse myelitis, enteric neuropathy, encephalitis and aseptic meningitis, although their incidence is <10%. The American Society of Clinical Oncology (ASCO) and the National Comprehensive Cancer Network (NCCN) guidelines on the management of irAEs suggest mild to high dose oral corticosteroids in the case of moderate to significant neurological toxicity, additional plasmapheresis or intravenous immunoglobulin may be required for the treatment of myasthenia and Guillain-Barre’ syndrome (Brahmer et al., 2018; Reid et al., 2021).

Although irAEs can sometimes be associated with mortality and significant lifelong morbidity (i.e., *de novo* insulin-dependent diabetes, persistent pituitary dysfunction, or immune-related inflammatory arthropathies), studies on irAEs are still limited in many clinical settings due to the rarity of specific events and absence of appropriate tissue material (Young et al., 2018; Head et al., 2019a). The clinical aspects of ICI-related toxicities are difficult to assess in the early stage, due to the relatively subtle or absent imaging changes. In this scenario, the early identification of patients who are more likely to develop irAEs before their clinical evidence, and their adequate monitoring especially when severe adverse effects occur becomes a challenge (Jia et al., 2020). Understanding the relationships between T-cell subsets, cytokines, and irAEs in larger cohorts may help to identify biomarkers for the early detection of immune toxicity, promoting the selection of optimal candidates for ICI therapy. In this regard, a sustained and early rise in tumor-specific CD8^+^ T-cell counts has been reported to be correlated with benefit achieved with ICIs. This means that a rise of specific T-cell subsets beyond a critical threshold as compared to pre-treatment values, or significant alterations between subsequent cycles, are likely predictive for irAEs (Naqash et al., 2019). Identification of biomarkers predictive of the onset of specific irAEs may help to reduce the risk of severe toxicities, treatment discontinuation, hence improving the cost-effectiveness of cancer immunotherapy (Xu et al., 2020).

There is a reciprocal interaction between cancer treatments and the gut microbiome. Of note, technological advances have recently allowed assessment of the composition and function of the microbiome in human health, and its role in various diseases (Joseph et al., 2020). There is pre-clinical evidence of increased levels of TNFα and IL-1β secreted by epithelial cells after co-incubation of fecal bacterial suspensions from irradiated compared with naïve mice. Significant alterations in the microbiota profile of cancer patients triggering radiation-induced disease, including mucositis, diarrhea and fatigue, have also been observed, with a subsequent reduction of the quality of life leading to treatment suspension (Zhu et al., 2017; Wu et al., 2019). Preclinical tumor mouse models able to mimic human autoimmune toxicities are needed to understand the irAE biology and mechanisms (Wei et al., 2018; Khan and Gerber, 2020). In the near future, the use of genetic information to predict responses to drugs is expected to provide more insight into the identification of patients more likely to experience severe irAEs (Berger and Mardis, 2018; Waldman et al., 2020).

The growing evidence of a possible immune-mediated *abscopal* tumor response to radiation out of the irradiated field makes it reasonable to ask whether radiation-induced sensitization of CD8^+^ T cells to self-antigens may also drive systemic ICI toxicity. In fact, the increased pro-immunogenic properties of ablative SBRT schedules *in vitro* might affect the spectrum and severity of irAEs (Hwang et al., 2018; Sha et al., 2020).

Diversification of the TCR repertoire, and the related expansion of the CD8^+^ T-cell population, may be the biological key in approaches combining RT plus immunotherapy to achieve maximal effectiveness while modulating, or if possible preventing, serious irAEs. The amplification of immune recognition and expansion of the cytotoxic T-cell compartment induced by the IFNɣ signaling axis may be another crosslink in combined strategies. Restoration of the optimal gut microbial composition, in turn, could improve the prevention of radiation-induced mucositis and enhance tumor killing, thus laying the foundation for successful combinations of RT and ICIs.

Given the new insights into the physiopathology of irAEs, which have been proving to be not just limited to autoreactive mechanisms, a close collaboration among clinical oncologists, radiation oncologists and organ specialists will be desirable. Evidence that dysimmune toxicity may be associated with the tumor response requires caution in the use of steroids and/or other immune suppressor drugs.

Further studies, randomized controlled trials are needed to understand the true impact of fractionation on toxicities of concurrent RT + ICIs.

Investigation on radiotherapy modulation of irAEs in both preclinical models and clinical settings may contribute to develop better strategies to prevent or manage toxicities without compromising treatment efficacy.

Improving our mechanistic understanding of the impact of ICIs on immune tolerance is an essential framework to ensure appropriate patients selection, in the context of patient-tailored treatment approaches, as well as to engineer alternative therapies that may guarantee a safe and effective antitumor immune response with minimal, or predictable, easily manageable side effects.

## References

[B1] Abolhassani A. R. Schuler G. Kirchberger M. C. Heinzerling L. (2019). C-Reactive Protein as an Early Marker of Immune-Related Adverse Events. J. Cancer Res. Clin. Oncol. 145 (10), 2625–2631. 10.1007/s00432-019-03002-1 31492984 PMC11810282

[B2] Abu-Sbeih H. Herrera L. N. Tang T. Altan M. Chaftari A. P. Okhuysen P. C. (2019). Impact of Antibiotic Therapy on the Development and Response to Treatment of Immune Checkpoint Inhibitor-Mediated Diarrhea and Colitis. J. Immunother. Cancer 7 (1), 242. 10.1186/s40425-019-0714-x 31488205 PMC6729015

[B3] Al-Qadami G. Van Sebille Y. Le H. Bowen J. (2019). Gut Microbiota: Implications for Radiotherapy Response and Radiotherapy-Induced Mucositis. Expert Rev. Gastroenterol. Hepatol. 13 (5), 485–496. 10.1080/17474124.2019.1595586 30907164

[B4] Alatrash G. Daver N. Mittendorf E. A. (2016). Targeting Immune Checkpoints in Hematologic Malignancies. Pharmacol. Rev. 68 (4), 1014–1025. 10.1124/pr.116.012682 27664133 PMC11060433

[B5] Albarel F. Castinetti F. Brue T. (2019). Management of Endocrine Disease: Immune Check point Inhibitors-Induced Hypophysitis. Eur. J. Endocrinol. 181 (3), R107–R118. 10.1530/EJE-19-0169 31311002

[B6] Anscher M. S. Arora S. Weinstock C. Lubitz R. Amatya A. Fiero M. (2020). Impact of Radiotherapy on Risk of Adverse Events in Patients Receiving Immunotherapy: A U.S. Food and Drug Administration Pooled Analysis. J. Clin. Oncol. 38 (15_Suppl. l), 3018. 10.1200/JCO.2020.38.15_suppl.3018

[B7] Ascierto M. L. Melero I. Ascierto P. A. (2015). Melanoma: From Incurable Beast to a Curable Bet. The success of Immunotherapy. Front. Oncol. 5, 152. 10.3389/fonc.2015.00152 26217587 PMC4500097

[B8] Atreya R. Mudter J. Finotto S. Müllberg J. Jostock T. Wirtz S. (2000). Blockade of Interleukin 6 Trans Signaling Suppresses T-Cell Resistance against Apoptosis in Chronic Intestinal Inflammation: Evidence in Crohn Disease and Experimental Colitis *In Vivo* . Nat. Med. 6 (5), 583–588. 10.1038/75068 10802717

[B9] Bang A. Schoenfeld J. D. (2019). Immunotherapy and Radiotherapy for Metastatic Cancers. Ann. Palliat. Med. 8 (3), 312–325. 10.21037/apm.2018.07.10 30180743

[B10] Bang A. Wilhite T. J. Pike L. R. G. Cagney D. N. Aizer A. A. Taylor A. (2017). Multicenter Evaluation of the Tolerability of Combined Treatment with PD-1 and CTLA-4 Immune Checkpoint Inhibitors and Palliative Radiation Therapy. Int. J. Radiat. Oncol. Biol. Phys. 98 (2), 344–351. 10.1016/j.ijrobp.2017.02.003 28463153

[B11] Barber F. D. (2019). Adverse Events of Oncologic Immunotherapy and Their Management. Asia Pac. J. Oncol. Nurs. 6 (3), 212–226. 10.4103/apjon.apjon_6_19 31259216 PMC6518984

[B12] Bartkowiak T. Jaiswal A. R. Ager C. R. Chin R. Chen C. H. Budhani P. (2018). Activation of 4-1BB on Liver Myeloid Cells Triggers Hepatitis via an Interleukin-27-Dependent Pathway. Clin. Cancer Res. 24 (5), 1138–1151. 10.1158/1078-0432.CCR-17-1847 29301830 PMC6752715

[B13] Berger M. F. Mardis E. R. (2018). The Emerging Clinical Relevance of Genomics in Cancer Medicine. Nat. Rev. Clin. Oncol. 15 (6), 353–365. 10.1038/s41571-018-0002-6 29599476 PMC6658089

[B14] Berman D. Parker S. M. Siegel J. Chasalow S. D. Weber J. Galbraith S. (2010). Blockade of Cytotoxic T-Lymphocyte Antigen-4 by Ipilimumab Results in Dysregulation of Gastrointestinal Immunity in Patients with Advanced Melanoma. Cancer Immun. 10, 11. 21090563 PMC2999944

[B15] Berner F. Bomze D. Diem S. Ali O. H. Fässler M. Ring S. (2019). Association of Checkpoint Inhibitor-Induced Toxic Effects with Shared Cancer and Tissue Antigens in Non-Small Cell Lung Cancer. JAMA Oncol. 5 (7), 1043–1047. 10.1001/jamaoncol.2019.0402 31021392 PMC6487908

[B16] Bomze D. Hasan Ali O. Bate A. Flatz L. (2019). Association between Immune-Related Adverse Events during Anti-PD-1 Therapy and Tumor Mutational burden. JAMA Oncol. 5 (11), 1633–1635. 10.1001/jamaoncol.2019.3221 31436791 PMC6707013

[B17] Brahmer J. R. Lacchetti C. Schneider B. J. Atkins M. B. Brassil K. J. Caterino J. M. (2018). Management of Immune-Related Adverse Events in Patients Treated with Immune Checkpoint Inhibitor Therapy: American Society of Clinical Oncology Clinical Practice Guideline. J. Clin. Oncol. 36 (17), 1714–1768. 10.1200/JCO.2017.77.6385 29442540 PMC6481621

[B18] Bulliard Y. Jolicoeur R. Windman M. Rue S. M. Ettenberg S. Knee D. A. (2013). Activating Fc γ Receptors Contribute to the Antitumor Activities of Immunoregulatory Receptor-Targeting Antibodies. J. Exp. Med. 210 (9), 1685–1693. 10.1084/jem.20130573 23897982 PMC3754864

[B19] Calles A. Aguado G. Sandoval C. Álvarez R. (2019). The Role of Immunotherapy in Small Cell Lung Cancer. Clin. Transl Oncol. 21 (8), 961–976. 10.1007/s12094-018-02011-9 30637710

[B20] Chaput N. Lepage P. Coutzac C. Soularue E. Le Roux K. Monot C. (2017). Baseline Gut Microbiota Predicts Clinical Response and Colitis in Metastatic Melanoma Patients Treated with Ipilimumab. Ann. Oncol. 28 (6), 1368–1379. 10.1093/annonc/mdx108 28368458

[B21] Citrin D. E. Mitchell J. B. (2017). Mechanisms of Normal Tissue Injury from Irradiation. Semin. Radiat. Oncol. 27 (4), 316–324. 10.1016/j.semradonc.2017.04.001 28865514 PMC5653270

[B22] Daly L. E. Power D. G. O'Reilly Á. Donnellan P. Cushen S. J. O'Sullivan K. (2017). The Impact of Body Composition Parameters on Ipilimumab Toxicity and Survival in Patients with Metastatic Melanoma. Br. J. Cancer 116, 310–317. 10.1038/bjc.2016.431 28072766 PMC5294486

[B23] Das R. Bar N. Ferreira M. Newman A. M. Zhang L. Bailur J. K. (2018). Early B Cell Changes Predict Autoimmunity Following Combination Immune Checkpoint Blockade. J. Clin. Invest. 128, 715–720. 10.1172/JCI96798 29309048 PMC5785243

[B24] De Felice F. Marchetti C. Palaia I. Musio D. Muzii L. Tombolini V. (2015). Immunotherapy of Ovarian Cancer: The Role of Checkpoint Inhibitors. J. Immunol. Res. 2015, 191832. 10.1155/2015/191832 26236750 PMC4508475

[B25] de Moel E. C. Rozeman E. A. Kapiteijn E. H. Verdegaal E. M. E. Grummels A. Bakker J. A. (2019). Autoantibody Development under Treatment with Immune-Checkpoint Inhibitors. Cancer Immunol. Res. 7 (1), 6–11. 10.1158/2326-6066.CIR-18-0245 30425107

[B26] Demaria S. Formenti S. C. (2012). Radiation as an Immunological Adjuvant: Current Evidence on Dose and Fractionation. Front. Oncol. 2, 153. 10.3389/fonc.2012.00153 23112958 PMC3481113

[B27] Dewan M. Z. Galloway A. E. Kawashima N. Dewyngaert J. K. Babb J. S. Formenti S. C. (2009). Fractionated but Not Single-Dose Radiotherapy Induces an Immune-Mediated Abscopal Effect when Combined with Anti-CTLA-4 Antibody. Clin. Cancer Res. 15 (17), 5379–5388. 10.1158/1078-0432.CCR-09-0265 19706802 PMC2746048

[B28] Diehl A. Yarchoan M. Hopkins A. Jaffee E. Grossman S. A. (2017). Relationships between Lymphocyte Counts and Treatment-Related Toxicities and Clinical Responses in Patients with Solid Tumors Treated with PD-1 Checkpoint Inhibitors. Oncotarget 8 (69), 114268–114280. 10.18632/oncotarget.23217 29371985 PMC5768402

[B29] Dubin K. Callahan M. K. Ren B. Khanin R. Viale A. Ling L. (2016). Intestinal Microbiome Analyses Identify Melanoma Patients at Risk for Checkpoint-Blockade-Induced Colitis. Nat. Commun. 7, 10391. 10.1038/ncomms10391 26837003 PMC4740747

[B30] Dubrot J. Milheiro F. Alfaro C. Palazón A. Martínez-Forero I. Perez-Gracia J. L. (2010). Treatment with Anti-CD137 mAbs Causes Intense Accumulations of Liver T Cells without Selective Antitumor Immunotherapeutic Effects in This Organ. Cancer Immunol. Immunother. 59 (8), 1223–1233. 10.1007/s00262-010-0846-9 20336294 PMC11030554

[B31] Eckert F. Gaipl U. S. Niedermann G. Hettich M. Schilbach K. Huber S. M. (2017). Beyond Checkpoint Inhibition - Immunotherapeutical Strategies in Combination with Radiation. Clin. Transl Radiat. Oncol. 2, 29–35. 10.1016/j.ctro.2016.12.006 29657997 PMC5893529

[B32] Eckert F. Schilbach K. Klumpp L. Bardoscia L. Sezgin E. C. Schwab M. (2018). Potential Role of CXCR4 Targeting in the Context of Radiotherapy and Immunotherapy of Cancer. Front. Immunol. 9, 3018. 10.3389/fimmu.2018.03018 30622535 PMC6308162

[B33] El Sabbagh R. Azar N. S. Eid A. A. Azar S. T. (2020). Thyroid Dysfunctions Due to Immune Checkpoint Inhibitors: A Review. Int. J. Gen. Med. 13, 1003–1009. 10.2147/IJGM.S261433 33177863 PMC7650809

[B34] El-Osta H. Shahid K. Mills G. M. Peddi P. (2016). Immune Checkpoint Inhibitors: The New Frontier in Non-Small-Cell Lung Cancer Treatment. Onco Targets Ther. 9, 5101–5116. 10.2147/OTT.S111209 27574451 PMC4993420

[B35] Elkrief A. Derosa L. Kroemer G. Zitvogel L. Routy B. (2019). The Negative Impact of Antibiotics on Outcomes in Cancer Patients Treated with Immunotherapy: A New Independent Prognostic Factor? Ann. Oncol. 30 (10), 1572–1579. 10.1093/annonc/mdz206 31268133

[B36] Esfahani K. Elkrief A. Calabrese C. Lapointe R. Hudson M. Routy B. (2020). Moving towards Personalized Treatments of Immune-Related Adverse Events. Nat. Rev. Clin. Oncol. 17 (8), 504–515. 10.1038/s41571-020-0352-8 32246128

[B37] Esfahani K. Meti N. Miller W. H. Jr Hudson M. (2019). Adverse Events Associated with Immune Checkpoint Inhibitor Treatment for Cancer. CMAJ 191 (2), E40–E46. 10.1503/cmaj.180870 30642824 PMC6333545

[B38] Esfahani K. Roudaia L. Buhlaiga N. Del Rincon S. V. Papneja N. Miller W. H. Jr (2020). A Review of Cancer Immunotherapy: from the Past, to the Present, to the Future. Curr. Oncol. 27 (Suppl. 2), S87–S97. 10.3747/co.27.5223 32368178 PMC7194005

[B39] Formenti S. C. Demaria S. (2009). Systemic Effects of Local Radiotherapy. Lancet Oncol. 10 (7), 718–726. 10.1016/S1470-2045(09)70082-8 19573801 PMC2782943

[B40] Friedlander P. Wood K. Wassmann K. Christenfeld A. M. Bhardwaj N. Oh W. K. (2018). A Whole-Blood RNA Transcript-Based Gene Signature Is Associated with the Development of CTLA-4 Blockade-Related Diarrhea in Patients with Advanced Melanoma Treated with the Checkpoint Inhibitor Tremelimumab. J. Immunother. Cancer 6 (1), 90. 10.1186/s40425-018-0408-9 30227886 PMC6145108

[B41] Fujisawa Y. Yoshino K. Otsuka A. Funakoshi T. Fujimura T. Yamamoto Y. (2017). Fluctuations in Routine Blood Count Might Signal Severe Immune-Related Adverse Events in Melanoma Patients Treated with Nivolumab. J. Dermatol. Sci. 88 (2), 225–231. 10.1016/j.jdermsci.2017.07.007 28736218

[B42] Fukihara J. Sakamoto K. Koyama J. Ito T. Iwano S. Morise M. (2019). Prognostic Impact and Risk Factors of Immune-Related Pneumonitis in Patients with Non-Small-Cell Lung Cancer Who Received Programmed Death 1 Inhibitors. Clin. Lung Cancer 20, 442–450. e444. 10.1016/j.cllc.2019.07.006 31446020

[B43] Gandhi S. J. Minn A. J. Vonderheide R. H. Wherry E. J. Hahn S. M. Maity A. (2015). Awakening the Immune System with Radiation: Optimal Dose and Fractionation. Cancer Lett. 368 (2), 185–190. 10.1016/j.canlet.2015.03.024 25799953

[B44] Gong J. Gerendash B. Dizman N. Khan A. Pal S. K. (2016). Advances in Treatment of Metastatic Renal Cell Carcinoma. Curr. Opin. Urol. 26 (5), 439–446. 10.1097/MOU.0000000000000319 27467136

[B45] Gudd C. L. C. Au L. Triantafyllou E. Shum B. Liu T. Nathwani R. (2021). Activation and Transcriptional Profile of Monocytes and CD8+ T Cells Are Altered in Checkpoint Inhibitor-Related Hepatitis. J. Hepatol. 75 (1), 177–189. 10.1016/j.jhep.2021.02.008 33631227

[B46] Haanen J. B. A. G. Carbonnel F. Robert C. Kerr K. M. Peters S. Larkin J. (2018). Management of Toxicities from Immunotherapy: ESMO Clinical Practice Guidelines for Diagnosis, Treatment and Follow-Up. Ann. Oncol. 29 (Suppl. 4), iv264–iv266. 10.1093/annonc/mdy162 29917046

[B47] Hasan Ali O. Berner F. Bomze D. Fässler M. Diem S. Cozzio A. (2019). Human Leukocyte Antigen Variation Is Associated with Adverse Events of Checkpoint Inhibitors. Eur. J. Cancer 107, 8–14. 10.1016/j.ejca.2018.11.009 30529903

[B48] Head L. Gorden N. Van Gulick R. Amato C. M. Frazer-Abel A. Robinson W. (2019). Biomarkers to Predict Immune-Related Adverse Events with Checkpoint Inhibitors. J. Clin. Oncol. 37 (Suppl. 8), 131. 10.1200/JCO.2019.37.8_suppl.131

[B49] Head L. Gorden N. Van Gulick R. Amato C. M. Frazer-Abel A. Robinson W. (2019). Biomarkers to Predict Immune-Related Adverse Events with Checkpoint Inhibitors. J. Clin. Oncol. 37 (8_Suppl. l), 131. 10.1200/jco.2019.37.8_suppl.131

[B50] Hodi F. S. O'Day S. J. McDermott D. F. Weber R. W. Sosman J. A. Haanen J. B. (2010). Improved Survival with Ipilimumab in Patients with Metastatic Melanoma. N. Engl. J. Med. 363 (8), 711–723. 10.1056/NEJMoa1003466 20525992 PMC3549297

[B51] Hofman P. (2019). Is the Onset of Adverse Effects of Immunotherapy Always Bad News for the Patients…?-Certainly Not!?-certainly Not. Ann. Transl Med. 7 (Suppl. 1), S5. 10.21037/atm.2019.01.14 31032286 PMC6462600

[B52] Hu H. Zakharov P. N. Peterson O. J. Unanue E. R. (2020). Cytocidal Macrophages in Symbiosis with CD4 and CD8 T Cells Cause Acute Diabetes Following Checkpoint Blockade of PD-1 in NOD Mice. Proc. Natl. Acad. Sci. U S A. 117 (49), 31319–31330. 10.1073/pnas.2019743117 33229539 PMC7733808

[B53] Hwang W. L. Pike L. R. G. Royce T. J. Mahal B. A. Loeffler J. S. (2018). Safety of Combining Radiotherapy with Immune-Checkpoint Inhibition. Nat. Rev. Clin. Oncol. 15 (8), 477–494. 10.1038/s41571-018-0046-7 29872177

[B54] Jagodinsky J. C. Harari P. M. Morris Z. S. (2020). The Promise of Combining Radiation Therapy with Immunotherapy. Int. J. Radiat. Oncol. Biol. Phys. 108 (1), 6–16. 10.1016/j.ijrobp.2020.04.023 32335187 PMC7442714

[B55] Jia X. H. Geng L. Y. Jiang P. P. Xu H. Nan K. J. Yao Y. (2020). The Biomarkers Related to Immune Related Adverse Events Caused by Immune Checkpoint Inhibitors. J. Exp. Clin. Cancer Res. 39 (1), 284. 10.1186/s13046-020-01749-x 33317597 PMC7734811

[B56] John-Aryankalayil M. Palayoor S. T. Cerna D. Simone C. B. 2nd Falduto M. T. Magnuson S. R. (2010). Fractionated Radiation Therapy Can Induce a Molecular Profile for Therapeutic Targeting. Radiat. Res. 174 (4), 446–458. 10.1667/RR2105.1 20726711

[B57] Johncilla M. Misdraji J. Pratt D. S. Agoston A. T. Lauwers G. Y. Srivastava A. (2015). Ipilimumab-associated Hepatitis: Clinicopathologic Characterization in a Series of 11 Cases. Am. J. Surg. Pathol. 39, 1075–1084. 10.1097/PAS.0000000000000453 26034866

[B58] Johnson D. B. Balko J. M. Compton M. L. Chalkias S. Gorham J. Xu Y. (2016). Fulminant Myocarditis with Combination Immune Checkpoint Blockade. N. Engl. J. Med. 375, 1749–1755. 10.1056/NEJMoa1609214 27806233 PMC5247797

[B59] Joseph N. T. Shankar S. R. Narasimhamurthy R. K. Rao S. B. S. Mumbrekar K. D. (2020). Bi-Directional Interactions between Microbiota and Ionizing Radiation in Head and Neck and Pelvic Radiotherapy - Clinical Relevance. Int. J. Radiat. Biol. 96 (8), 961–971. 10.1080/09553002.2020.1770361 32420768

[B60] Kachikwu E. L. Iwamoto K. S. Liao Y. P. DeMarco J. J. Agazaryan N. Economou J. S. (2011). Radiation Enhances Regulatory T Cell Representation. Int. J. Radiat. Oncol. Biol. Phys. 81 (4), 1128–1135. 10.1016/j.ijrobp.2010.09.034 21093169 PMC3117954

[B61] Khan S. Gerber D. E. (2020). Autoimmunity, Checkpoint Inhibitor Therapy and Immune-Related Adverse Events: A Review. Semin. Cancer Biol. 64, 93–101. 10.1016/j.semcancer.2019.06.012 31330185 PMC6980444

[B62] Khan S. Khan S. A. Luo X. Fattah F. J. Saltarski J. Gloria-McCutchen Y. (2018). Immune Dysregulation in Cancer Patients Developing Immune-Related Adverse Events. Br. J. Cancer 120 (1), 63–68. 10.1038/s41416-018-0155-1 30377338 PMC6325132

[B63] Kilger E. Buehler A. Woelfing H. Kumar S. Kaeser S. A. Nagarathinam A. (2011). BRI2 Protein Regulates β-Amyloid Degradation by Increasing Levels of Secreted Insulin-Degrading Enzyme (IDE). J. Biol. Chem. 286 (43), 37446–37457. 10.1074/jbc.M111.288373 21873424 PMC3199491

[B64] Kobayashi T. Iwama S. Yasuda Y. Okada N. Tsunekawa T. Onoue T. (2018). Patients with Antithyroid Antibodies Are Prone to Develop Destructive Thyroiditis by Nivolumab: a Prospective Study. J. Endocr. Soc. 2, 241–251. 10.1210/js.2017-00432 29600292 PMC5836529

[B65] Kuehn H. S. Ouyang W. Lo B. Deenick E. K. Niemela J. E. Avery D. T. (2014). Immune Dysregulation in Human Subjects with Heterozygous Germline Mutations in CTLA4. Science 345 (6204), 1623–1627. 10.1126/science.1255904 25213377 PMC4371526

[B66] Kurimoto C. Inaba H. Ariyasu H. Iwakura H. Ueda Y. Uraki S. (2020). Predictive and Sensitive Biomarkers for Thyroid Dysfunctions during Treatment with Immune-Checkpoint Inhibitors. Cancer Sci. 111, 1468–1477. 10.1111/cas.14363 32086984 PMC7226278

[B67] Lacouture M. E. Wolchok J. D. Yosipovitch G. Kähler K. C. Busam K. J. Hauschild A. (2014). Ipilimumab in Patients with Cancer and the Management of Dermatologic Adverse Events. J. Am. Acad. Dermatol. 71, 161–169. 10.1016/j.jaad.2014.02.035 24767731

[B68] Läubli H. Balmelli C. Bossard M. Pfister O. Glatz K. Zippelius A. (2015). Acute Heart Failure Due to Autoimmune Myocarditis under Pembrolizumab Treatment for Metastatic Melanoma. J. Immunother. Cancer 3, 11. 10.1186/s40425-015-0057-1 25901283 PMC4404586

[B69] Lim S. Y. Lee J. H. Gide T. N. Menzies A. M. Guminski A. Carlino M. S. (2019). Circulating Cytokines Predict Immune-Related Toxicity in Melanoma Patients Receiving Anti-PD-1-Based Immunotherapy. Clin. Cancer Res. 25 (5), 1557–1563. 10.1158/1078-0432.CCR-18-2795 30409824

[B70] Luke J. J. Lemons J. M. Karrison T. G. Pitroda S. P. Melotek J. M. Zha Y. (2018). Safety and Clinical Activity of Pembrolizumab and Multisite Stereotactic Body Radiotherapy in Patients with Advanced Solid Tumors. J. Clin. Oncol. 36 (16), 1611–1618. 10.1200/JCO.2017.76.2229 29437535 PMC5978468

[B71] Lynch D. Murphy A. (2016). The Emerging Role of Immunotherapy in Colorectal Cancer. Ann. Transl Med. 4 (16), 305. 10.21037/atm.2016.08.29 27668225 PMC5009029

[B72] Martin A. M. Cagney D. N. Catalano P. J. Alexander B. M. Redig A. J. Schoenfeld J. D. (2018). Immunotherapy and Symptomatic Radiation Necrosis in Patients with Brain Metastases Treated with Stereotactic Radiation. JAMA Oncol. 4 (8), 1123–1124. 10.1001/jamaoncol.2017.3993 29327059 PMC5885198

[B73] Martins F. Sofiya L. Sykiotis G. P. Lamine F. Maillard M. Fraga M. (2019). Adverse Effects of Immune-Checkpoint Inhibitors: Epidemiology, Management and Surveillance. Nat. Rev. Clin. Oncol. 16 (9), 563–580. 10.1038/s41571-019-0218-0 31092901

[B74] Michot J. M. Bigenwald C. Champiat S. Collins M. Carbonnel F. Postel-Vinay S. (2016). Immune-related Adverse Events with Immune Checkpoint Blockade: a Comprehensive Review. Eur. J. Cancer 54, 139–148. 10.1016/j.ejca.2015.11.016 26765102

[B75] Mihic-Probst D. Reinehr M. Dettwiler S. Kolm I. Britschgi C. Kudura K. (2020). The Role of Macrophages Type 2 and T-Regs in Immune Checkpoint Inhibitor Related Adverse Events. Immunobiology 225 (5), 152009. 10.1016/j.imbio.2020.152009 32962812

[B76] Milling L. Zhang Y. Irvine D. J. (2017). Delivering Safer Immunotherapies for Cancer. Adv. Drug Deliv. Rev. 114, 79–101. 10.1016/j.addr.2017.05.011 28545888 PMC5647831

[B77] Moskovitz J. Moy J. Ferris R. L. (2018). Immunotherapy for Head and Neck Squamous Cell Carcinoma. Curr. Oncol. Rep. 20 (2), 22. 10.1007/s11912-018-0654-5 29502288 PMC5835060

[B78] Muraro E. Furlan C. Avanzo M. Martorelli D. Comaro E. Rizzo A. (2017). Local High-Dose Radiotherapy Induces Systemic Immunomodulating Effects of Potential Therapeutic Relevance in Oligometastatic Breast Cancer. Front. Immunol. 8, 1476. 10.3389/fimmu.2017.01476 29163540 PMC5681493

[B79] Naqash A. R. Appah E. Yang L. V. Muzaffar M. Marie M. A. Mccallen J. D. (2019). Isolated Neutropenia as a Rare but Serious Adverse Event Secondary to Immune Checkpoint Inhibition. J. Immunother. Cancer 7 (1), 169. 10.1186/s40425-019-0648-3 31277704 PMC6612131

[B80] Ngiow S. F. Teng M. W. Smyth M. J. (2013). A Balance of Interleukin-12 and -23 in Cancer. Trends Immunol. 34 (11), 548–555. 10.1016/j.it.2013.07.004 23954142

[B81] Nishimura H. Nose M. Hiai H. Minato N. Honjo T. (1999). Development of Lupus-Like Autoimmune Diseases by Disruption of the PD-1 Gene Encoding an ITIM Motif-Carrying Immunoreceptor. Immunity 11 (2), 141–151. 10.1016/s1074-7613(00)80089-8 10485649

[B82] Nishimura H. Okazaki T. Tanaka Y. Nakatani K. Hara M. Matsumori A. (2001). Autoimmune Dilated Cardiomyopathy in PD-1 Receptor-Deficient Mice. Science 291 (5502), 319–322. 10.1126/science.291.5502.319 11209085

[B83] Niu L. Strahotin S. Hewes B. Zhang B. Zhang Y. Archer D. (2007). Cytokine-mediated Disruption of Lymphocyte Trafficking, Hemopoiesis, and Induction of Lymphopenia, Anemia, and Thrombocytopenia in Anti-CD137-Treated Mice. J. Immunol. 178 (7), 4194–4213. 10.4049/jimmunol.178.7.4194 17371976 PMC2770095

[B84] Oh D. Y. Cham J. Zhang L. Fong G. Kwek S. S. Klinger M. (2017). Immune Toxicities Elicted by CTLA-4 Blockade in Cancer Patients Are Associated with Early Diversification of the T-Cell Repertoire. Cancer Res. 77 (6), 1322–1330. 10.1158/0008-5472.CAN-16-2324 28031229 PMC5398199

[B85] Okiyama N. Tanaka R. (2017). Varied Immuno-Related Adverse Events Induced by Immune-Check point Inhibitors - Nivolumab-Associated Psoriasiform Dermatitis Related with Increased Serum Level of Interleukin-6. Nihon Rinsho Meneki Gakkai Kaishi 40 (2), 95–101. 10.2177/jsci.40.95 28603207

[B86] Osorio J. C. Ni A. Chaft J. E. Pollina R. Kasler M. K. Stephens D. (2017). Antibody-Mediated Thyroid Dysfunction during T-Cell Checkpoint Blockade in Patients with Non-Small-Cell Lung Cancer. Ann. Oncol. 28, 583–589. 10.1093/annonc/mdw640 27998967 PMC5834017

[B87] Overacre-Delgoffe A. E. Chikina M. Dadey R. E. Yano H. Brunazzi E. A. Shayan G. (2017). Interferon-γ Drives Treg Fragility to Promote Anti-Tumor Immunity. Cell 169, 1130–e11. 10.1016/j.cell.2017.05.005 28552348 PMC5509332

[B88] Parker S. M. Zainib M. Mattes M. Amin N. (2018). Multi-Institutional Report on Toxicities from Combined Radiation and Nivolumab. J. Clin. Oncol. 36, 39. 10.1200/JCO.2018.36.5_suppl.39 PMC6128090 30202808

[B89] Patel S. A. Minn A. J. (2018). Combination Cancer Therapy with Immune Checkpoint Blockade: Mechanisms and Strategies. Immunity 48 (3), 417–433. 10.1016/j.immuni.2018.03.007 29562193 PMC6948191

[B90] Petrelli F. Ardito R. Borgonovo K. Lonati V. Cabiddu M. Ghilardi M. (2018). Haematological Toxicities with Immunotherapy in Patients with Cancer: A Systematic Review and Meta-Analysis. Eur. J. Cancer 103, 7–16. 10.1016/j.ejca.2018.07.129 30196108

[B91] Peverelli E. Mantovani G. Lania A. G. Spada A. (2014). cAMP in the Pituitary: An Old Messenger for Multiple Signals. J. Mol. Endocrinol. 52 (1), R67–R77. 10.1530/JME-13-0172 24049068

[B92] Pistillo M. P. Fontana V. Morabito A. Dozin B. Laurent S. Carosio R. (2019). Soluble CTLA-4 as a Favorable Predictive Biomarker in Metastatic Melanoma Patients Treated with Ipilimumab: An Italian Melanoma Intergroup Study. Cancer Immunol. Immunother. 68 (1), 97–107. 10.1007/s00262-018-2258-1 30311027 PMC11028053

[B93] Porcu M. De Silva P. Solinas C. Battaglia A. Schena M. Scartozzi M. (2019). Immunotherapy Associated Pulmonary Toxicity: Biology Behind Clinical and Radiological Features. Cancers (Basel) 11 (3), 305. 10.3390/cancers11030305 PMC6468855 30841554

[B94] Powell J. D. Pollizzi K. N. Heikamp E. B. Horton M. R. (2012). Regulation of Immune Responses by mTOR. Annu. Rev. Immunol. 30, 39–68. 10.1146/annurev-immunol-020711-075024 22136167 PMC3616892

[B95] Rakhmanov M. Keller B. Gutenberger S. Foerster C. Hoenig M. Driessen G. (2009). Circulating CD21low B Cells in Common Variable Immunodeficiency Resemble Tissue Homing, Innate-Like B Cells. Proc. Natl. Acad. Sci. U S A. 106 (32), 13451–13456. 10.1073/pnas.0901984106 19666505 PMC2726348

[B96] Rapisuwon S. Izar B. Batenchuk C. Avila A. Mei S. Sorger P. (2019). Exceptional Response and Multisystem Autoimmune-Like Toxicities Associated with the Same T Cell Clone in a Patient with Uveal Melanoma Treated with Immune Checkpoint Inhibitors. J. Immunother. Cancer 7 (1), 61. 10.1186/s40425-019-0533-0 30832716 PMC6399858

[B97] Reid P. D. Cifu A. S. Bass A. R. (2021). Management of Immunotherapy-Related Toxicities in Patients Treated with Immune Checkpoint Inhibitor Therapy. JAMA 325 (5), 482–483. 10.1001/jama.2020.17308 33528524

[B98] Rojas M. Restrepo-Jiménez P. Monsalve D. M. Pacheco Y. Acosta-Ampudia Y. Ramírez-Santana C. (2018). Molecular Mimicry and Autoimmunity. J. Autoimmun. 95, 100–123. 10.1016/j.jaut.2018.10.012 30509385

[B99] Schindler K. Harmankaya K. Kuk D. Mangana J. Michielin O. Hoeller C. (2014). Correlation of Absolute and Relative Eosinophil Counts with Immune- Related Adverse Events in Melanoma Patients Treated with Ipilimumab. J. Clin. Oncol. 32 (Suppl. 15), 9096. [abstract]. 10.1200/jco.2014.32.15_suppl.9096

[B100] Schwab C. Gabrysch A. Olbrich P. Patiño V. Warnatz K. Wolff D. (2018). Phenotype, Penetrance, and Treatment of 133 Cytotoxic T-Lymphocyte Antigen 4-insufficient Subjects. J. Allergy Clin. Immunol. 142 (6), 1932–1946. 10.1016/j.jaci.2018.02.055 29729943 PMC6215742

[B101] Schweizer C. Schubert P. Rutzner S. Eckstein M. Haderlein M. Lettmaier S. (2020). Prospective Evaluation of the Prognostic Value of Immune-Related Adverse Events in Patients with Non-Melanoma Solid Tumour Treated with PD-1/pd-L1 Inhibitors Alone and in Combination with Radiotherapy. Eur. J. Cancer 140, 55–62. 10.1016/j.ejca.2020.09.001 33045663

[B102] Selby M. J. Engelhardt J. J. Quigley M. Henning K. A. Chen T. Srinivasan M. (2013). Anti-CTLA-4 Antibodies of IgG2a Isotype Enhance Antitumor Activity through Reduction of Intratumoral Regulatory T Cells. Cancer Immunol. Res. 1 (1), 32–42. 10.1158/2326-6066.CIR-13-0013 24777248

[B103] Sha C. M. Lehrer E. J. Hwang C. Trifiletti D. M. Mackley H. B. Drabick J. J. (2020). Toxicity in Combination Immune Checkpoint Inhibitor and Radiation Therapy: A Systematic Review and Meta-Analysis. Radiother. Oncol. 151, 141–148. 10.1016/j.radonc.2020.07.035 32717359

[B104] Shahabi V. Berman D. Chasalow S. D. Wang L. Tsuchihashi Z. Hu B. (2013). Gene Expression Profiling of Whole Blood in Ipilimumab-Treated Patients for Identification of Potential Biomarkers of Immune-Related Gastrointestinal Adverse Events. J. Transl Med. 11, 75. 10.1186/1479-5876-11-75 23521917 PMC3637501

[B105] Shaverdian N. Beattie J. Thor M. Offin M. Shepherd A. F. Gelblum D. Y. (2020). Safety of Thoracic Radiotherapy in Patients with Prior Immune-Related Adverse Events from Immune Checkpoint Inhibitors. Ann. Oncol. 31 (12), 1719–1724. 10.1016/j.annonc.2020.09.016 33010460 PMC8284745

[B106] Simpson T. R. Li F. Montalvo-Ortiz W. Sepulveda M. A. Bergerhoff K. Arce F. (2013). Fc-Dependent Depletion of Tumor-Infiltrating Regulatory T Cells Co-Defines the Efficacy of Anti-CTLA-4 Therapy against Melanoma. J. Exp. Med. 210 (9), 1695–1710. 10.1084/jem.20130579 23897981 PMC3754863

[B107] Stamatouli A. M. Quandt Z. Perdigoto A. L. Clark P. L. Kluger H. Weiss S. A. (2018). Collateral Damage: Insulin-Dependent Diabetes Induced with Checkpoint Inhibitors. Diabetes 67, 1471–1480. 10.2337/dbi18-0002 29937434 PMC6054443

[B108] Suresh K. Naidoo J. Zhong Q. Xiong Y. Mammen J. de Flores M. V. (2019). The Alveolar Immune Cell Landscape Is Dysregulated in Checkpoint Inhibitor Pneumonitis. J. Clin. Invest. 129 (10), 4305–4315. 10.1172/JCI128654 31310589 PMC6763233

[B109] Sutton C. E. Lalor S. J. Sweeney C. M. Brereton C. F. Lavelle E. C. Mills K. H. (2019). Interleukin-1 and IL-23 Induce Innate IL-17 Production from Gammadelta T Cells, Amplifying Th17 Responses and Autoimmunity. Immunity 31 (2), 331–341. 10.1016/j.immuni.2009.08.001 19682929

[B110] Tahir S. A. Gao J. Miura Y. Blando J. Tidwell R. S. S. Zhao H. (2019). Autoimmune Antibodies Correlate with Immune Checkpoint Therapy-Induced Toxicities. Proc. Natl. Acad. Sci. U S A. 116 (44), 22246–22251. 10.1073/pnas.1908079116 31611368 PMC6825284

[B111] Tarhini A. A. Zahoor H. Lin Y. Malhotra U. Sander C. Butterfield L. H. (2015). Baseline Circulating IL-17 Predicts Toxicity while TGF-Β1 and IL-10 Are Prognostic of Relapse in Ipilimumab Neoadjuvant Therapy of Melanoma. J. Immunother. Cancer 3, 39. 10.1186/s40425-015-0081-1 26380086 PMC4570556

[B112] Toi Y. Sugawara S. Sugisaka J. Ono H. Kawashima Y. Aiba T. (2019). Profiling Preexisting Antibodies in Patients Treated with Anti-PD-1 Therapy for Advanced Non-Small Cell Lung Cancer. JAMA Oncol. 5 (3), 376–383. 10.1001/jamaoncol.2018.5860 30589930 PMC6439838

[B113] Tsai C. S. Chen F. H. Wang C. C. Huang H. L. Jung S. M. Wu C. J. (2007). Macrophages from Irradiated Tumors Express Higher Levels of iNOS, Arginase-I and COX-2, and Promote Tumor Growth. Int. J. Radiat. Oncol. Biol. Phys. 68 (2), 499–507. 10.1016/j.ijrobp.2007.01.041 17398016

[B114] Tsai M. H. Cook J. A. Chandramouli G. V. DeGraff W. Yan H. Zhao S. (2007). Gene Expression Profiling of Breast, Prostate, and Glioma Cells Following Single versus Fractionated Doses of Radiation. Cancer Res. 67 (8), 3845–3852. 10.1158/0008-5472.CAN-06-4250 17440099

[B115] Valpione S. Pasquali S. Campana L. G. Piccin L. Mocellin S. Pigozzo J. (2018). Sex and Interleukin-6 Are Prognostic Factors for Autoimmune Toxicity Following Treatment with Anti-CTLA4 Blockade. J. Transl Med. 16 (1), 94. 10.1186/s12967-018-1467-x 29642948 PMC5896157

[B116] Vanneste B. G. L. Van Limbergen E. J. Dubois L. Samarska I. V. Wieten L. Aarts M. J. B. (2020). Immunotherapy as Sensitizer for Local Radiotherapy. OncoImmunology 9 (1), 1832760. 10.1080/2162402X.2020.1832760 33194319 PMC7605354

[B117] Waldman A. D. Fritz J. M. Lenardo M. J. (2020). A Guide to Cancer Immunotherapy: From T Cell Basic Science to Clinical Practice. Nat. Rev. Immunol. 20 (11), 651–668. 10.1038/s41577-020-0306-5 32433532 PMC7238960

[B118] Wang D. Quiros J. Mahuron K. Pai C. C. Ranzani V. Young A. (2018). Targeting EZH2 Reprograms Intratumoral Regulatory T Cells to Enhance Cancer Immunity. Cell Rep 23, 3262–3274. 10.1016/j.celrep.2018.05.050 29898397 PMC6094952

[B119] Wang Y. Deng W. Li N. Neri S. Sharma A. Jiang W. (2018). Combining Immunotherapy and Radiotherapy for Cancer Treatment: Current Challenges and Future Directions. Front. Pharmacol. 9, 185. 10.3389/fphar.2018.00185 29556198 PMC5844965

[B120] Ward F. J. Dahal L. N. Wijesekera S. K. Abdul-Jawad S. K. Kaewarpai T. Xu H. (2013). The Soluble Isoform of CTLA-4 as a Regulator of T-Cell Responses. Eur. J. Immunol. 43 (5), 1274–1285. 10.1002/eji.201242529 23400950

[B121] Weber J. S. Dummer R. de Pril V. Lebbé C. Hodi F. S. (2013). Patterns of Onset and Resolution of Immune-Related Adverse Events of Special Interest with Ipilimumab: Detailed Safety Analysis from a Phase 3 Trial in Patients with Advanced Melanoma. Cancer 119 (9), 1675–1682. 10.1002/cncr.27969 23400564

[B122] Wei S. C. Duffy C. R. Allison J. P. (2018). Fundamental Mechanisms of Immune Checkpoint Blockade Therapy. Cancer Discov. 8 (9), 1069–1086. 10.1158/2159-8290.CD-18-0367 30115704

[B123] Weinmann S. C. Pisetsky D. S. (2019). Mechanisms of Immune-Related Adverse Events during the Treatment of Cancer with Immune Checkpoint Inhibitors. Rheumatology (Oxford) 58 (Suppl. 7), vii59–vii67. 10.1093/rheumatology/kez308 31816080 PMC6900913

[B124] Wennerberg E. Vanpouille-Box C. Bornstein S. Yamazaki T. Demaria S. Galluzzi L. (2017). Immune Recognition of Irradiated Cancer Cells. Immunol. Rev. 280 (1), 220–230. 10.1111/imr.12568 29027232 PMC5659195

[B125] Wu X. Zhang T. Chen X. Ji G. Zhang F. (2019). Microbiota Transplantation: Targeting Cancer Treatment. Cancer Lett. 452, 144–151. 10.1016/j.canlet.2019.03.010 30905818

[B126] Xu Y. Fu Y. Zhu B. Wang J. Zhang B. (2020). Predictive Biomarkers of Immune Checkpoint Inhibitors-Related Toxicities. Front. Immunol. 11, 2023. 10.3389/fimmu.2020.02023 33123120 PMC7572846

[B127] Yamauchi I. Yasoda A. Matsumoto S. Sakamori Y. Kim Y. H. Nomura M. (2019). Incidence, Features, and Prognosis of Immune-Related Adverse Events Involving the Thyroid Gland Induced by Nivolumab. PLoS ONE 14, e0216954. 10.1371/journal.pone.0216954 31086392 PMC6516638

[B128] Young A. Quandt Z. Bluestone J. A. (2018). The Balancing Act between Cancer Immunity and Autoimmunity in Response to Immunotherapy. Cancer Immunol. Res. 6 (12), 1445–1452. 10.1158/2326-6066.CIR-18-0487 30510057 PMC6281171

[B129] Zhang T. Yu H. Ni C. Zhang T. Liu L. Lv Q. (2017). Hypofractionated Stereotactic Radiation Therapy Activates the Peripheral Immune Response in Operable Stage I Non-small-cell Lung Cancer. Sci. Rep. 7 (1), 4866. 10.1038/s41598-017-04978-x 28687760 PMC5501824

[B130] Zhu X. X. Yang X. J. Chao Y. L. Zheng H. M. Sheng H. F. Liu H. Y. (2017). The Potential Effect of Oral Microbiota in the Prediction of Mucositis during Radiotherapy for Nasopharyngeal Carcinoma. EBioMedicine 18, 23–31. 10.1016/j.ebiom.2017.02.002 28216066 PMC5405060

